# Supplementary material for: Social sentiment segregation: Evidence from Twitter and Google Trends in Chile during the COVID-19 dynamic quarantine strategy
Source: PLoS One. 2021 Jul 13;16(7):e0254638. doi: 10.1371/journal.pone.0254638 (PMC8277056; doi:10.1371/journal.pone.0254638)
Supplement: S1 Table — This table presents population information for the 120 Chilean municipalities with more than 13,000 inhabitants. Municipalities are sorted from low to high, according to their MPI. (PDF) [file pone.0254638.s001.pdf]

Table S1: This table presents population information for the 120 Chilean municipalities with more than 13,000 inhabitants. Municipalities are sorted, from low to high, according to their MPI.

Municipalities	Inhabitants	MPI
Vitacura	85.384	0,1%
Las Condes	294.838	0,2%
Providencia	142.079	0,4%
Ñuñoa	208.237	0,9%
La Reina	92.787	1,0%
Punta Arenas	131.592	2,1%
Concón	42.152	2,4%
Maipú	521.627	2,6%
Coyhaique	57.818	2,8%
Lo Barnechea	105.833	2,8%
Lampa	102.034	3,6%
Talagante	74.237	3,7%
Quinta Normal	110.026	3,7%
Renca	147.151	3,7%
Villa Alemana	126.548	3,7%
Santiago	404.495	4,1%
Paine	72.759	4,2%
Peñalolén	241.599	4,4%
Iquique	191.468	4,4%
Limache	46.121	4,4%
La Florida	366.916	4,5%
Calama	165.731	4,6%
San Ramón	82.900	4,6%
La Granja	116.571	4,8%
San Miguel	107.954	4,8%
Peñaflor	90.201	4,9%
Santa Cruz	37.855	5,0%
Antofagasta	361.873	5,1%
Melipilla	13.467	5,2%
Viña del Mar	334.248	5,2%
San Joaquín	45.547	5,2%
Quilpué	151.708	5,3%
Quintero	31.923	5,3%
Huechuraba	98.671	5,6%
Quilicura	210.410	5,7%
Ovalle	111.272	5,7%
Lo Prado	96.249	5,8%
Estación Central	147.041	5,8%
Puerto Varas	44.578	5,9%
San José de Maipo	94.492	6,0%
Pedro Aguirre Cerda	101.174	6,2%
Copiapó	153.937	6,3%
Colina	146.207	6,4%
Cerrillos	80.832	6,5%
La Cisterna	90.119	6,6%
Lo Espejo	98.804	6,7%
Cartagena	22.738	6,9%
Recoleta	157.851	6,9%
Los Andes	66.708	7,0%
Graneros	33.437	7,0%
Valparaíso	296.655	7,1%
San Vicente	46.766	7,1%
Machalí	52.505	7,2%
Tomé	54.946	7,3%
Puente Alto	568.106	7,3%
Conchalí	126.955	7,4%
Macul	116.534	7,5%
San Antonio	58.825	7,5%
Constitución	46.068	7,5%
Cerro Navia	132.622	7,6%
Valdivia	166.080	7,6%
Concepción	223.574	7,7%
Alto Hospicio	108.375	7,8%
Talcahuano	151.749	7,9%
Talca	220.357	8,0%
Buin	96.614	8,0%
San Felipe	43.269	8,2%
Pudahuel	230.293	8,2%
Isla de Maipo	36.219	8,3%
Arica	221.364	8,3%
Independencia	100.281	8,5%
Osorno	161.460	8,5%
Penco	47.367	8,6%
Chiguayante	85.938	8,7%
El Monte	35.923	8,7%
Padre Hurtado	63.250	8,8%
Vallenar	51.917	8,8%
Teno	28.921	9,2%
San Bernardo	91.350	9,4%
El Bosque	162.505	9,6%
Curicó	149.136	9,9%
Quillota	90.517	9,9%
San Fernando	76.844	10,1%
Castro	43.807	10,1%
La Serena	221.054	10,2%
Temuco	282.415	10,4%
Tocopilla	25.186	10,4%
Hualpén	91.773	10,5%
Rancagua	241.774	10,6%
Maule	49.721	11,0%
Linares	93.602	11,3%
Coronel	116.262	11,4%
Puerto Montt	245.902	11,4%
Chillán	184.739	11,7%
Illapel	30.848	12,0%
San Pedro de la Paz	131.808	12,2%
Curanilahue	32.288	12,3%
Villarrica	55.478	12,5%
Molina	45.976	13,1%
Lebu	25.522	13,2%
La Unión	38.036	13,3%
Coquimbo	227.730	13,4%
San Carlos	301.313	13,8%
La Pintana	177.335	14,1%
Ancud	38.991	14,5%
Mulchén	29.627	14,8%
Angol	53.262	15,6%
Calera	50.554	15,8%
San Clemente	53.024	16,7%
La Ligua	35.390	16,7%
Lautaro	38.013	16,8%
Parral	41.637	16,9%
Nacimiento	26.315	17,4%
Los Ángeles	202.331	17,4%
Lota	43.535	18,1%
Chillán Viejo	30.907	19,1%
Padre Las Casas	76.126	19,6%
Cauquenes	40.441	20,0%
San Javier	73.973	20,0%
Victoria	34.182	22,5%
