# Supplementary material for: Social sentiment segregation: Evidence from Twitter and Google Trends in Chile during the COVID-19 dynamic quarantine strategy
Source: PLoS One. 2021 Jul 13;16(7):e0254638. doi: 10.1371/journal.pone.0254638 (PMC8277056; doi:10.1371/journal.pone.0254638)
Supplement: Attachment — Submitted filename: PONE-D-21-01436.pdf [file pone.0254638.s003.pdf]

Referee Report – PONE-D-21-01436

"Twitter and Google Trends Sentiment in a highly segregated Economy: The COVID-19 Case in Chile"

This is an insightful study of stock market and pandemic related sentiment reactions to lockdown announcements. Moreover, it manages to highlight the role of socioeconomic characteristics as a confounding factor. Therefore, I would like to congratulate the authors on the very topical and relevant paper. I greatly enjoyed reading the paper and the results could be impactful and informative for the journal's readership. However, some methodological choices and caveats require further consideration.

These are my comments that I would like to ask the authors to address:

Major comments:

1. The authors cut off the municipalities to include at 13,000 inhabitants, but it is not very clear why exactly they truncate the data there. The readers would benefit from a justification of this choice. Moreover, a sensitivity analysis with respect to this assumption seems highly recommendable.
2. In the same vein, the authors choose to focus on the 15 wealthiest municipalities based on the MPI. The main reason for doing so appears to be somewhat arbitrary: matching the 12% of population that belong to the ABC1 socioeconomic sentiment.
   a. First, it seems highly likely that such matching `of the ABC1´ is masking substantial differences in practices. For example, the 12% of population included in the 15 selected municipalities will in all likelihood also include a substantial share of people that are not in the top segment. Therefore, making any direct inference from the results relating to the ABC1 misleading.
   b. Second, given the limited underpinning for selecting just these 15 municipalities, the authors should provide sensitivity analysis on this assumption. For instance, to what extent do their conclusions change when increasing or decreasing the number of included municipalities?
   c. Third, would it not be more insightful to also make direct use of the quantitative information captured in the MPI, e.g. by including interaction terms instead of a more arbitrary subsample selection? Currently, the methodological setup disregards all the relative information captured by the MPI.
3. For the construction of their variables of interest (cf. equations 5 and 10), the authors employ the median over the previous five days for normalization. This is laudable. However, one significant issue is not accounted for: day-of-week effects, neither in the construction of the variables nor the estimation. Nonetheless, they are significant for, for example, social media usage and may therefore cloud the results.
4. The regression results reported in Tables 2-4 report significance levels out of the ordinary for economic and financial publications. Using anything higher than 0.1 for the * (such as, 0.15 in the case of the authors) is advised against. Sticking to the standard of "* p<0.1, ** p<0.05 and *** p<0.01" is highly recommended for several reasons. First, it eases comparison across studies and prevents misleading readers. Second, drawing any conclusion based on a p-value larger than 0.1 is stretching the results beyond what can be reasonably expected. Consequently, I would insist that the authors adjust their reported statistics and conclusions accordingly. Plenty of their conclusions do withstand this. In addition, I ask the authors to

clarify the levels of significance reported in Table 1 as well, since they do not seem to be reported.

5. Finally, the large majority of the authors´ conclusions – excluding maybe their inferences based on the mere correlations in Figures 4-6 – are based on samples of only 25 observations. Even with an adjusted estimator such as that of Cribari-Neto this still casts major doubt on the robustness of the results.
   Overall, this goes to the core of a methodological choice made. Whereas the analysis starts from rich daily data for March-July, the chosen methods boil all of this down into just 25 observations. It goes to wonder whether there is not a more robust estimation approach available. In particular, it seems that the main advantage of the approach chosen by the authors is the announcement specific analysis portrayed in Table 1. Nevertheless, little is done with this detail in the subsequent part of the paper, since all these announcement dates are then pooled.
   Therefore, I suggest that the authors reconsider the estimation approaches in Tables 2-4. For example, since no further announcement specific inferences are made there, a heterogeneous effect difference-in-difference method could be fitted to the data. Moreover, it could just as well be used to test the additional hypotheses of the authors. Most importantly, it would enable using the more detailed sample of daily data and thus more robust inferences.

Minor comments:

1. What type of capitalization is used for the title? Why is “highly segregated” not capitalized. In any case, I am not fully convinced by the added value of this qualification in the title, as the socioeconomic distinction used in the paper is not a measure of segregation per se.
2. In the abstract:
   a. In the first sentence, the word “government” can be dropped, since it is implied by the reference to the health authority.
   b. Further, “observed stock market abnormal returns” should be replaced by “observed abnormal stock market returns”.
3. On line 9, “the country´s announcement” should be rephrased. Only a country´s government can make an announcement.
4. In lines 19-21, the authors refer to a decline in informal employment. What statistic and source are the authors referring to here, i.e. how exactly is this measured? In its current phrasing, the evidence seems only anecdotal.
5. In lines 24-26, the authors draw conclusions on the evolution of the degree of “information dissemination” based on the number of related tweets. However, to my understanding, a tweet does not by default contain information. In fact, it could just as well be spreading disinformation. Therefore, the authors´ conclusion seems to be too strong.
6. On multiple occasions, the authors write “Tweeter” instead of “Twitter”, including in the conclusion. Please correct this.
7. The text contains three different spellings of lockdown: lock-downs (line 37), lock downs (line 141) and lockdowns (144). Please converge on one.
8. On line 96 of the manuscript the verb should be plural not singular (i.e. “show”) in order to be consistent with the rest of the text.
9. The conclusion in lines 402-403 should be qualified. Among the users of Google queries, there seems to be no socioeconomic segregation “as measured by a truncation of municipalities based on the MPI” (cf. Major comment 2).
