# Supplementary material for: Social sentiment segregation: Evidence from Twitter and Google Trends in Chile during the COVID-19 dynamic quarantine strategy
Source: PLoS One. 2021 Jul 13;16(7):e0254638. doi: 10.1371/journal.pone.0254638 (PMC8277056; doi:10.1371/journal.pone.0254638)
Supplement: Attachment — Submitted filename: Response to Reviewers.pdf [file pone.0254638.s005.pdf]

Santiago, Chile, April 14th, 2021

Dear,
Stefan Cristian Gherghina, Academic Editor,
PLOS ONE

We would like to thank you for your letter on 1 March 2021 with the editorial decision of the manuscript 'Twitter and Google Trends Sentiment in a highly segregated Economy: The COVID-19 Case in Chile' (Díaz,F.,Henríquez, P.A.).

We have completed the revision of the manuscript according to the comments of the Academic Editor (AE) and the Reviewers.

In this letter, the comments (C) by the AE and Reviewers are reproduced and a detailed answer (A) is given concerning the changes made in the manuscript.

AE:
C1: The paper approaches a very topical subject, but many drawbacks befall. For instance, the discussion regarding prior literature should be expanded, the quantitative framework should be improved, whereas the practical implications of the research should be noticed. Besides, the English language should be improved.

A1: Thank you for your comment. The literature review has been expanded. To keep the manuscript at a reasonable length, in Section 2, Table 1, we present a brief review of recent works related to sentiment analysis.

Regarding the quantitative methods, we have performed sensitivity analyses and conducted robustness checks to confirm the validity of our results. Specifically, in section 4.2, Table 3, Panel B, we present results for the cumulative abnormal returns of the IGPA index observed upon government announcements, considering an alternative sorting of municipalities, based on *Municipal Income*. We obtain similar results, both in magnitude and statistical significance, to those obtained for the original *MPI* sorting. We have also added results obtained for different wealth cohorts of the population, and not only for the *ABC1* segment of the population, as suggested by

some Reviewers. A similar sensitivity analysis have been performed for our sentiment proxies in tables 4 and 5 in Section 4.3.

Also regarding our quantitative framework, and as an alternative approach to analyze the impact of quarantine announcements on market sentiment, we consider the suggestion of one of the Reviewers and perform a panel data regression similar to the *Difference in Difference (DiD)* methodology, which allows us to take advantage of the panel structure of our data, increasing considerably the sample size for estimation. Results are presented in Table 6.

With respect to the practical implications of our work, these are now discussed in the Introduction and in the Conclusions.

Finally, as regards to language, the revised document has been proofread by a professional proofreading service.

REVIEWER 1:

C1: This paper analyzes the impact of changes in the quarantine status of Chilean municipalities on stock market returns, trends in Google searches, and sentiment as inferred by activity on Twitter. The main results are that events in which richer municipalities enter (exit) quarantine are associated with negative (positive) reactions on the stock market and on sentiment (as inferred via Twitter activity), and that increases in the total amount of population under quarantine are associated to increases in COVID-related search activity on Google.

I think the paper combines an important question with an interesting empirical setting and carefully collected data. However, I have some major concerns on the measurement, the empirical analysis, and the interpretation of the results.

A1: Thank you for your comment. We believe you have understood the nature of the paper. We have performed sensitivity analyses and conducted robustness checks to confirm the validity of our results. We discuss the implications of our work both in the Introductions and in the Conclusions of the revised version.

C2: 1) The interpretation of the results is pushed in the direction of inferring degrees of "segregation" among users of Google search or Twitter. I find this interpretation not fully supported and, in any case, not central to

the message of the paper. If the question of the paper is to determine the reaction of Google and Twitter users to changes in the quarantine status of different municipalities, why shall we try to address the unrelated question of users' selection into those platforms?

A2: Thank you for your comment. Originally, the aim of the paper was to analyze sentiment responses and stock markets reactions to government announcements, considering the number of people affected by the corresponding lockdowns, with no regards to their socioeconomic status. That is probably why the gist of the original document was not clear. Following your comment, we substantially changed the way the paper is written so that it now emphasizes the socioeconomic segregation among social media (Twitter and Google Trends) users. Also, thanks to the suggestions of other reviewers, we provide additional evidence that further substantiates the existence of the above-mentioned phenomenon.

C3: 2) Similarly, stock market segregation is mentioned in the abstract and the paper, but never properly defined. The statement that stock market segregation mirrors socioeconomic segregation, and that this result can be derived from the finding on stock market returns should be clarified or better explained. In any case, I think this statement doesn't belong neither to the abstract nor to the introduction. Related to this point, I would de-emphasize the claim in the last paragraph of the Literature Review and the last paragraph before the conclusions.

A3: Thank you for your comment. We realize that the statement that stock market segregation mirrors socioeconomic segregation was improper in the article's context. The relevant point is that social sentiment segregation mirrors stock market segregation. This has been changed accordingly in the abstract and conclusions. Also, following your suggestion, we de-emphasize this claim as a central finding in our work and explain that the stock market analysis was performed mainly to validate the proposed *SES* sorting among municipalities.

C4: 3) The authors use the Multidimension Poverty Index (MPI) as a proxy for socio-economic status. Why not using more standard measures such as average per capita income? Related to this point, how did the authors select the 12% threshold to define municipalities in the "wealthy" group? I think

it is important to show at least some robustness around these measures and definitions.

A4: Thank you very much for this comment. As a robustness check, we consider an alternative sorting, based on *Municipal Income*. Municipal income includes all sources of financing available to municipalities; collection of business licenses, income from land and property taxes, payment of road taxes, fines collected by the municipality, as well as transfers from the Central Government. We applied this sorting to the analysis of stock market reactions in Panel B of Table 3. We obtain similar results to those obtained for the *MPI* sorting. In any case, our preferred measure is still the *MPI*. According to the results of a US representative survey of adult Twitter users carried out by the Pew Research Center, Twitter users do not only have higher incomes, but are also more highly educated and differ from the broader population on some key social issues which are more likely to be captured by the broader scope of the *MPI*.

As regards to the 12% threshold, this figure corresponds to the proportion of people belonging to the $ABC1$ socioeconomic segment for 2018, according to the Association of Market Researchers and Public Opinion of Chile. Following your suggestion, in section 4, Tables 3, 4, and 5, we perform a sensitivity analysis and present our results for different wealth cohorts, not only for the *ABC1* segment of the population.

C5: 4) How are the (-1,0) and the (-1,+1) windows defined? Is 0 defined as the day of the announcement? It is critical to properly justify the transformation of the outcome variables in “cumulative” terms between t1 and t2. It is unclear why cumulating is the right transformation (instead, for example, of taking the difference between t2 and t1). In general, event-study settings should isolate the impact of the event by comparing a pre-event outcome with a post-event outcome, but my understanding of the empirical setting is that the dependent variables are averages of the pre- and post-event outcomes.

A5: Thank you for your comment. The (-1,0) and the (-1,+1) windows are now properly defined in section 3.2, line 225.

Regarding the transformation of the outcome variables in “cumulative” terms inside the event windows, this is the standard practice in the Event Studies methodology for stock returns in finance, since it is likely that there is quite a bit of variation of the returns across days within the event window.

As leakage of information occurs through time, the accumulated abnormal returns are considered better measures of the effect of firm-specific events. The use of cumulative outcomes is popular in other areas of management too. For instance, Skiera et al (2017) (**link**) investigate what should be the dependent variable in marketing-related event studies and always rely on cumulative transformations of the alternative measures they consider. In any case, following the suggestion of another Reviewer, and as an alternative approach to analyze the impact of quarantine announcements on market sentiment, we consider a panel data regression similar to the *Difference in Difference (DiD)* methodology, in which the *Abnormal Sentiment Activity* index and the *Abnormal Search Volume Activity* index, which are not accumulated outcomes, are used. The variables of interest are compared between groups before (day -1), after (day +1) and on the day of the announcement (day 0), so in this way we hope to be addressing your concern that we should by comparing a pre-event outcome with a post-event outcome.

C6: 5) What are the “Sentiment Controls” used in Equation (13)? Are they the same as the “Sentiment Score” defined in Equation (8)? Why are they included in Equation (13)? Since sentiments are likely to be themselves affected by quarantine decisions, including them as a control might make inference worse. I have the same concern about Equations (14) and (15), where CARt, an outcome in Equation (13), here appears as a control.

A6: Thank you for your insightful comment. The “Sentiment Controls” included in Eq. (13) in the original version of the document were, in fact, the sentiment proxies used later on in the sentiment analysis. In the revised version of our work, we do not include these controls in Eq. (5). As you suggest, the inclusion of such controls do not contribute to the overall message of the paper and might result confusing for the reader, in particular for the understanding of causality issues. Furthermore, as we point out in (A3) in response to one of your comments, we have de-emphasized the results of the stock market analysis as a central finding in our work and explain that it was performed to validate the ranking of municipalities in which our sentiment analysis is based on. In any case, when originally included as controls, these variables turned out to be statistically insignificant.

In the revised version of the paper, when analyzing the cumulative abnormal sentiment responses to government announcements, in Eq. (14) and Eq. (15), we keep the IGPA *CAR*s as a control variable to make sure that

sentiment responses can be explained by quarantine announcements on top of the effect that the economic situation, as reflected in stock market performance, might itself have on social sentiment. We also include now, as an additional control, the *Stringency Index*, to take into account the potential effect that the toughness of the containment measures might have on social sentiment. A similar strategy is followed when we carry out the panel data estimation in section 4.3.

C7: 6) Although the paper is certainly understandable, the quality of the writing is sometimes not as good as it could be. There are multiple typos (e.g. Twitter is occasionally spelled "Tweeter"), and some sentences that should be rephrased (e.g. "are statistically insignificant from 0" I believe should be "are statistically insignificant").

A7: Thank you, this has been changed as suggested. the revised document has been proofread by a professional proofreading service.

REVIEWER 2:
C1: This is an insightful study of stock market and pandemic related sentiment reactions to lockdown announcements. Moreover, it manages to highlight the role of socioeconomic characteristics as a confounding factor. Therefore, I would like to congratulate the authors on the very topical and relevant paper. I greatly enjoyed reading the paper and the results could be impactful and informative for the journal's readership. However, some methodological choices and caveats require further consideration.

These are my comments that I would like to ask the authors to address:

A1: Thank you very much. We believe you have understood the nature of the paper. We truly believe that this revised version is significantly better than the original submission.

C2: The authors cut off the municipalities to include at 13,000 inhabitants, but it is not very clear why exactly they truncate the data there. The readers would benefit from a justification of this choice. Moreover, a sensitivity analysis with respect to this assumption seems highly recommendable.

A2: Thank you very much for this comment. The 12% threshold corre-

sponds to the proportion of people belonging to the $ABC1$ socioeconomic segment in 2018, according to the Association of Market Researchers and Public Opinion of Chile. Following your suggestion, we perform a sensitivity analysis and present our results for different wealth cohorts, not only for the *ABC1* segment.

C3: In the same vein, the authors choose to focus on the 15 wealthiest municipalities based on the MPI. The main reason for doing so appears to be somewhat arbitrary: matching the 12% of population that belong to the ABC1 socioeconomic sentiment.
a. First, it seems highly likely that such matching 'of the ABC1 is masking substantial differences in practices. For example, the 12% of population included in the 15 selected municipalities will in all likelihood also include a substantial share of people that are not in the top segment. Therefore, making any direct inference from the results relating to the ABC1 misleading.
b. Second, given the limited underpinning for selecting just these 15 municipalities, the authors should provide sensitivity analysis on this assumption. For instance, to what extent do their conclusions change when increasing or decreasing the number of included municipalities?
c. Third, would it not be more insightful to also make direct use of the quantitative information captured in the MPI, e.g. by including interaction terms instead of a more arbitrary subsample selection? Currently, the methodological setup disregards all the relative information captured by the MPI.

A3: Thank you for your comments. We will attempt to address them one by one:

(a) You are entirely correct. There is undoubtedly socioeconomic heterogeneity within municipalities. However, as mentioned in the revised version of the paper, municipalities are the minor administrative division in Chile. The health authorities impose quarantines so that confinement affects the whole universe of inhabitants of each municipality. Accordingly, even if we had a more refined socioeconomic segmentation of people inside municipalities, that we don't, we would not be able to use it in our analysis.

(b) As mentioned in our response (A2), we performed the suggested sensitivity analysis, finding further support not only for socioeconomic segregation in social media (particularly for Twitter), but also for a similar phenomenon

in stock market reactions to quarantine announcements. Results are presented in Tables 3, 4, and 5.

(c) Your intuition is correct, since it is always better to take advantage of the whole variability of a measure rather than any democratization of such variable. However, the *MPI* is constant for each municipality in our sample period. Accordingly, including such an interaction term in either Eq 14 or Eq 15, would be like multiplying the value of $\Delta Population_{it}$ by a constant value MPI_i, for each municipality i.

C4: For the construction of their variables of interest (cf. equations 5 and 10), the authors employ the median over the previous five days for normalization. This is laudable. However, one significant issue is not accounted for: day-of-week effects, neither in the construction of the variables nor the estimation. Nonetheless, they are significant for, for example, social media usage and may therefore cloud the results.

A4: Thanks for your comment. Figure 1 and 2 show the Box plots for the day-of-the-week effects for both the Sentiment Score and the Average Search Volume Index. According to this figures, the median of both sentiment proxies is nearly constant throughout the week.

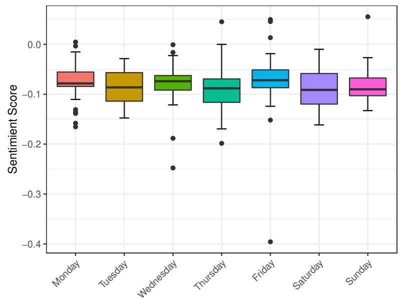

Figure 1: Day-of-week effects at the Sentiment Score.

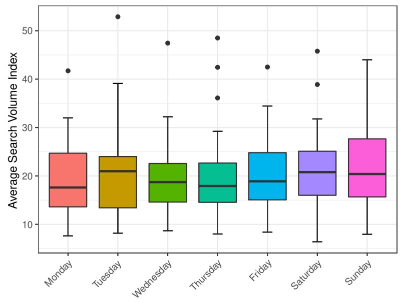

Figure 2: Day-of-week effects at the Average Search Volume Index.

C5: The regression results reported in Tables 2-4 report significance levels out of the ordinary for economic and financial publications. Using anything higher than 0.1 for the * (such as, 0.15 in the case of the authors) is advised

against. Sticking to the standard of "* $p < 0.1$, ** $p < 0.05$ and *** $p < 0.01$" is highly recommended for several reasons. First, it eases comparison across studies and prevents misleading readers. Second, drawing any conclusion based on a p-value larger than 0.1 is stretching the results beyond what can be reasonably expected. Consequently, I would insist that the authors adjust their reported statistics and conclusions accordingly. Plenty of their conclusions do withstand this. In addition, I ask the authors to clarify the levels of significance reported in Table 1 as well, since they do not seem to be reported.

A5: Thank you for your comment. We fixed significance levels as you requested, keeping significance level at the 15% level denoted by "+". In any case, most of our results feature significance levels between the 10% and the 1% level and these are the results that are now stressed in the paper. Regarding the missing levels of significance reported in Table 1 in the original manuscript, they are now properly reported in Table 2.

C6: Finally, the large majority of the authors conclusions – excluding maybe their inferences based on the mere correlations in Figures 4-6 – are based on samples of only 25 observations. Even with an adjusted estimator such as that of Cribari-Neto this still casts major doubt on the robustness of the results. Overall, this goes to the core of a methodological choice made. Whereas the analysis starts from rich daily data for March-July, the chosen methods boil all of this down into just 25 observations. It goes to wonder whether there is not a more robust estimation approach available. In particular, it seems that the main advantage of the approach chosen by the authors is the announcement specific analysis portrayed in Table 1. Nevertheless, little is done with this detail in the subsequent part of the paper, since all these announcement dates are then pooled. Therefore, I suggest that the authors reconsider the estimation approaches in Tables 2-4. For example, since no further announcement specific inferences are made there, a heterogeneous effect difference-in-difference method could be fitted to the data. Moreover, it could just as well be used to test the additional hypotheses of the authors. Most importantly, it would enable using the more detailed sample of daily data and thus more robust inferences.

A6: Thank you very much for your insightful comment. As you suggest, given the panel structure of our data, in which municipalities with different socioeconomic characteristics alternate frequently their lockdown status

, a natural way to proceed is to consider a *Difference in Difference (DiD)* approach for analysis. In our case, we have different treatment timings for different municipalities, a setup that is sometimes referred to as a *staggered DiD* model. However, strictly speaking, a standard *DiD* estimation is not feasible in our case. The standard *DiD* estimator is defined as the difference in average outcome in the treatment group, before and after treatment, minus the difference in average outcome in the control group, before and after treatment. But, in our case, we only observe the outcomes of interest, the social sentiment proxies, for the population as a whole and not for different segments. In other words, for each day t in our sample period, we only observe a single value of ASA_t or $ASVA_t$.

Despite the fact that we observe a unique value of the sentiment indexes for both the treatment and control groups, it is still possible to compute an estimator in the spirit of a *DiD* estimator. To see this, let y_t be the observable outcome, common to both groups, and consider a linear interaction model. The definition of the variables is presented in detail in section 3.5 of the revised document.

$$y_t = \alpha_1 + \sum_{l=-j}^{l=+j} \delta_l D_{it}^{(j)} + \sum_{l=-j}^{l=+j} \beta_l D_{it}^{(j)} \cdot W_i + \gamma W_i + \mu_{it} \tag{1}$$

Suppose that we are interested in the value that the outcome variable takes the day after the announcement day, $j = 1$. The expected value of y for a wealthy municipality that was announced to be quarantined at $t = t^*$ is:

$$E[y_{t^*+1}|\textit{wealthy and quarantined}] = \alpha_1 + \delta_{+1} + \beta_{+1} + \gamma \tag{2}$$

For a wealthy municipality that was not quarantined at $t = t^*$, the expected value of y is given by:

$$E[y_{t^*+1}|\textit{wealthy and not quarantined}] = \alpha_1 + \gamma \tag{3}$$

The expected difference in y_{t^*+1} between wealthy municipalities that are confined and not confined is therefore $\delta_{+1}+\beta_{+1}$. For non wealthy and confined municipalities, the expected value of y is given by:

$$E[y_{t^*+1}|\textit{not wealthy and quarantined}] = \alpha_1 + \delta_{+1} \tag{4}$$

And for not wealthy and not confined municipalities, the expected value of y is:

$$E[y_{t^*+1} | not\ wealthy\ and\ not\ quarantined] = \alpha_1 \quad (5)$$

The expected difference in y_{t^*+1} between confined and not confined, non wealthy municipalities, is therefore δ_{+1}. The parameter β_{+1} is then the average difference in y_{t^*+1} between the effects of being confined for wealthy municipalities and for non wealthy municipalities, the day after the quarantine announcement, where the average is taken over all days after a government announcement is made. Intuitively, this is similar to the event study methodology, but considering *event time* rather than *calendar time*. In this way, following your suggestion, we are able to take advantage of more detailed sample of daily data and thus to make more robust inferences. Results are presented in Table 6, with a sample size of 325 observations. The results of this approach are summarized in Fig. 8. More importantly, the result of this analysis confirm the effects documented in Tables 4 and 5 of the revised version of the paper.

C7: What type of capitalization is used for the title? Why is "highly segregated" not capitalized. In any case, I am not fully convinced by the added value of this qualification in the title, as the socioeconomic distinction used in the paper is not a measure of segregation per se.

A7: Thanks for you comment. You are completely right. Following your suggestion, we have updated the title to: "Social Sentiment Segregation: Evidence from Twitter and Google Trends in Chile during the COVID-19 Dynamic Quarantine Strategy".

C8: In the abstract:
a. In the first sentence, the word "government" can be dropped, since it is implied by the reference to the health authority.
b. Further, "observed stock market abnormal returns" should be replaced by "observed abnormal stock market returns".

A8: Thanks for your comment. The abstract has been changed according to your and other Reviewers' suggestions.

C9: On line 9, "the country s announcement" should be rephrased. Only a country s government can make an announcement.

A9: Thanks for your comment. This has been changed.

C10: In lines 19-21, the authors refer to a decline in informal employment. What statistic and source are the authors referring to here, i.e. how exactly is this measured? In its current phrasing, the evidence seems only anecdotal.

A10: Thanks for you comment. This information is reported by the National Statistics Institute (Instituto Nacional de Estadísticas, INE, www.ine.cl). We include the respective reference in the revised version of the paper (line 18).

C11: In lines 24-26, the authors draw conclusions on the evolution of the degree of "information dissemination" based on the number of related tweets. However, to my understanding, a tweet does not by default contain information. In fact, it could just as well be spreading disinformation. Therefore, the authors conclusion seems to be too strong.

A11: Thanks for you comment. This text has been changed to "The amount of information disseminated through social networks has experienced levels rarely seen. Kumar et al [5] report how Twitter has emerged as a critical tool for communicating the effect of this crisis and report that during its early stages, there was a COVID-19-related tweet every 45 ms. According to these authors, "A social media pandemic has preceded the disease pandemic, stirring a diversified spectrum of emotions"".

C12: On multiple occasions, the authors write "Tweeter" instead of "Twitter", including in the conclusion. Please correct this.

A12: Done

C13: The text contains three different spellings of lockdown: lock-downs (line 37), lock downs (line 141) and lockdowns (144). Please converge on one.

A13: Done

C14: On line 96 of the manuscript the verb should be plural not singular

(i.e. "show") in order to be consistent with the rest of the text.

A14: Done

C15: The conclusion in lines 402-403 should be qualified. Among the users of Google queries, there seems to be no socioeconomic segregation "as measured by a truncation of municipalities based on the MPI" (cf. Major comment 2).

A15: Thank you for your comment. In the revised version of the paper, in line 576, now it reads "Based on the evidence presented in Table 5, among the users of Google queries, there seems to be no socioeconomic segregation, as measured by a truncation of municipalities based on the *MPI*."

REVIEWER 3:

C1: Dear authors The corrections comments are based on the manuscript you were followed.
Concern 1: The justification behind this manuscript is weak and the problem is not valid.

A1: Thank you for your comment. We have thoroughly revised the Abstract, Introduction, and Conclusions of the paper to provide a clear motivation for our research question, emphasizing the novelty of our empirical approaches and the opportunity that the *dynamic quarantine* scheme constitutes for our work. We have also tried to place our work properly in the current literature, with its contributions and limitations.

C2: Concern 2: The strategies below would emphasize the novelty of your findings.
• Highlight the gaps in the Introduction section and mention how your study is going to address any/ some of the gaps
• In the presented Discussion, discuss the findings of the previous studies and specifically mention what new observation or insight was generated through your study results.
• In the Conclusion section, clearly, mention how your study advances the knowledge in the field

A2: Thank you for your comments. In the Introduction and the Conclusions,

we now emphasize how our work contributes to the existing literature and how our results can help policymakers. We highlight our work in the literature concerned with the causal effect between lockdowns and the population's overall well-being during the pandemic. This topic is attracting interest from both academicians and governments. Furthermore, in the Conclusions, we explain how our empirical approach, which hinges on the socioeconomic heterogeneity of Chilean municipalities and the dynamic features of the sanitary strategy, allows us to identify the socioeconomic status of social media users. This task has proven to be challenging to achieve in the literature of hidden types.

C3: Concern 3: Reorganize the abstract to conclude:
(a) The overall purpose of the study and the research problems you investigated.
(b) The basic design of the study.
(c) Major findings or trends found as a result of the study.
(d) A brief summary of your interpretations and conclusions.

A3: Thank you for your comment. We have completely reorganized the abstract according to your suggestions.

C4: Concern 4: The introduction needs to clarify the (1) motivation, (2) challenges, (3) contribution, (4) objectives, and (5) significance/implication. All the information (should be) presented in sequence idea.

A4: Thank you very much for your comment. We have completely reorganized the Introduction according to your suggestions. We believe that it provides now a much clearer and more comprehensive picture of our work.

C5: Concern 5: In literature review section, the comparative analysis should be pointed to testify that this study is more advanced than others. The comparative need to be illustrated through table consist of desire information among this study and others studies.

A5: Thanks for your comment. We have included Table 1 to emphasize the place of our work in the related recent literature.

C6: Concern 6: The authors mentioned the contribution in Literature re-

view Section (Our study also contributes to the literature on the socioeconomic status inference of social media hidden user characteristics, one of the most active fields in information retrieval. In this area, the following references are worth mentioning.). I think these confusing readers.

A6: Thank you for your comment. According to your suggestion, we have rephrased the beginning of that paragraph in line 140 of the revised version.

C7: Concern 7: Remove the duplicated sentences among sections. Summarize the article as much as possible.

A7: Thank you for your comment. We have tried to improve the article's writing style, trying to keep it as brief as we can. However, we have been suggested to add alternative empirical methodologies and perform sensitivity analyses and robustness checks, which have extended the paper's length.

C8: Concern 8: write the conclusion and consider the following comments:
- Highlight your analysis and reflect only the important points for the whole paper.
- Mention the benefits
- Mention the implication in the last of this section.

A8: Thank you for your comment. We have completely reorganized the Conclusion according to your suggestions. Furthermore, we now also discuss our work's limitations and how our results must be interpreted in light of these limitations.

C9: Concern 9: The paper has language and grammar issues. Carefully revised the paper by [a native English speaker]/[a professional language editing service] to improve the grammars. Please, carefully review the manuscript to resolve these issues.

A9: Thank you, this has been changed as suggested. The revised document has been proofread by a professional proofreading service..

REVIEWER 4:
C1: Please include more info about sentiment analysis in abstract

A1: Thank you for your suggestion. As you will notice, we have thoroughly revised the general structure of the paper to provide a clear motivation for our research question, emphasizing the novelty of our empirical approaches. We have attempted to reconcile the suggestion of all Reviewers. In this sense, to keep a reasonable length for the Abstract and to meet yours and other Reviewers suggestions, in Section 3.3 we explain in detail the logic and methods for the construction of our sentiment proxies.

C2: please indicate about the novelty of this work in comparison with other Sentiment analysis work

A2: Thanks for your comment. We have included Table 1 to emphasize the place of our work in the related recent literature.

C3: in your first two paragraphs, u did not include a single reference till the third paragraph, include more references in the first two paragraphs.

A3: Thank you for your comment. We have thoroughly revised the Introduction of the paper to provide a better motivation for our work, including, as you suggest, more references to place it properly in the current literature.

C4: before including about the Covid-19 and economy, please first discuss about the Covid-19 from medical causalities and death and statistics, then u can start talking about economy.

A4: Thank you for your comment. Your suggestion has been addressed in the revised version of the paper.

C5: include more recent studies related to Covid-19 and sentiment analysis like the following

Sentiment analysis of nationwide lockdown due to COVID 19 outbreak: Evidence from India

Sentiment analysis and its applications in fighting COVID-19 and infectious diseases: A systematic review

Sentiment Analysis of COVID-19 tweets by Deep Learning Classifiers—A study to show how popularity is affecting accuracy in social media

Twitter sentiment analysis on worldwide COVID-19 outbreaks

A5: Thank you for this suggestion. We have incorporated the above mentioned references in Table 1.

C6: introduce a section for preprocessing and discuss your preprocessing and its issues

A6: Thank you for your comment. As you will notice, we have reorganized the paper in many ways. The prepossessing and construction of our sentiment proxies is now presented in Section 3.3. Limitations regarding our sentiment proxies and related methods are presented in the Conclusions.

C7: introduce two sections for theoretical and practical significance of your work and indicate how your work is different from others

A7: Thank you for your comment. In the Introduction and the Conclusions, we now emphasize how our work contributes to the existing literature and how our results can help both academicians and policymakers. We highlight our work in the literature concerned with the causal effect between lockdowns and the population's overall well-being during the pandemic. In the Conclusions, we explain how our empirical approach, which hinges on the socioeconomic heterogeneity of Chilean municipalities and the dynamic features of the sanitary strategy, allows us to identify social media users' socioeconomic status. Finally, we have included Table 1 in the Literature Review section to emphasize our work in the related recent literature.

REVIEWER 5:

C1: The manuscript technically sound, and the data support the conclusions. Authors should explain some of the results. I have some doubts about the methodology and some robustness check should be done.

A1: Thank you very much. We have attempted to address all your suggestions.

C2: 1 Overwiew
This paper analyses social media and stock market reactions to the government announcements made by the Chilean health authority in the context of the COVID-19 pandemic. Taking advantage of the heterogeneity in the size and socioeconomic characteristics of the country's municipalities, the authors document a socioeconomic segregation phenomenon, both in the stock market and among Twitter users. The main findings can be summarized as follows: First, they provide evidence that observed stock market abnormal returns can be explained by the number of people from the wealthiest segment of the population that enters or exits confinement. Second, social sentiment based on Twitter tends to be more pessimistic, the higher the number of the wealthiest population that is quarantined.

2 Comments
Overall I enjoyed reading the paper and I consider the topic to be relevant, although certain aspects need to be improved. I have several suggestions which are aimed at clarifying the story the author is telling. These are not in any particular order.

A2: Thank you very much. We have tried to address all your concerns and include your suggestions. We believe that this revised version is significantly better than the original submission.

C3: 2.1- The proposed Google Sentiment measure is constructed using 19 terms. In general, queries based on keywords are language-specific and subject to ambiguity. The authors should clarify why using keywords instead of topics

A3: Thank you very much for your insightful comment. You are right, there might be ambiguity in the terms used to construct our proposed Google Sentiment measure. However, even if there is ambiguity in the search of pandemic related words, this goes against the effect we intent to quantify. If the search intensity of key words that are subject to ambiguity do not change around government announcements (because they are in fact ambiguous), the statistic in Eq 9 in the revised version of the paper would be downward biased:

$$ASVA_t = ln\left(\frac{ASVI_t}{med\{ASVI_{t-1},\ldots,ASVI_{t-5}\}}\right) \tag{9}$$

If a specific word is not related to the pandemic, its intensity search value would not change around government announcements. This word would not add to the value of the *Average Search Volume Intensity* index, $ASVI_t$. Consider the example in Table 1 below, in which we are considering only three words. Day t is the day of the government announcement. In the first case, there is no ambiguity. The search intensity of all three words changes (increases) upon the government announcement and $ASVA_t = 0.3$. In case 2, the first word is ambiguous, in the sense that its search intensity does not increase upon government announcements (instead of increasing to 98, as in Case 1, it gets a value close to the values observed in the previous days, 77, colored in red). In this case, $ASVA_t = 0.27$. With two ambiguous words, $ASVA_t = 0.14$, and with three ambiguous words $ASVA_t = 0.01$.

Given the economic and statistical significance of the observed effects presented in Table 2 of the revised version of the paper, it seems that ambiguity is not a serious problem. Also, of the 19 words, we believe that only 4 (corona, propagación, brote, restricción) can be considered ambiguous.

C4: 2.2- Related to the previous point, the authors argue that "the higher the value of the ASV in a given day t, the higher the population's attention to the outbreak of the Coronavirus on that day". However, this argument is not taking into the relationship between intensity and severity. Should each query be labelled as positive or negative? The authors should clarify this point

A4: Thank you for this insightful comment. Regarding the relationship between intensity and severity, this was not taken into account in the original version of the paper. Following your suggestion, we now include in all our empirical specifications related to sentiment the *Stringency Index*, to control for the effect that policy responses to the pandemic might have on market sentiment (details about this index are provided in the revised version of the paper on page 16). It ranges from 1 to 100 and records the strictness of lockdown style policies implemented by governments. As long as governments impose more strict lockdown policies when the pandemic status is more se-

**Case 1: No ambiguous words**					
Day	Word 1	Word 2	Word 3	ASVI	ASVA
t-5	70	25	40	45.0	
t-4	75	19	35	43.0	
t-3	72	22	33	42.3	
t-2	81	18	41	46.7	
t-1	79	24	39	47.3	
t	98	81	91	90.0	0.30
**Case 2: 1 ambiguous word**					
Day	Word 1 (Ambiguous)	Word 2	Word 3	ASVI	ASVA
t-5	70	25	40	45.0	
t-4	75	19	35	43.0	
t-3	72	22	33	42.3	
t-2	81	18	41	46.7	
t-1	79	24	39	47.3	
t	77	81	91	83.0	0.27
**Case 3: 2 ambiguous words**					
Day	Word 1 (Ambiguous)	Word 2 (Ambiguous)	Word 3	ASVI	ASVA
t-5	70	25	40	45.0	
t-4	75	19	35	43.0	
t-3	72	22	33	42.3	
t-2	81	18	41	46.7	
t-1	79	24	39	47.3	
t	77	20	91	62.7	0.14
**Case 4: 3 ambiguous words**					
Day	Word 1 (Ambiguous)	Word 2 (Ambiguous)	Word 3 (Ambiguous)	ASVI	ASVA
t-5	70	25	40	45.0	
t-4	75	19	35	43.0	
t-3	72	22	33	42.3	
t-2	81	18	41	46.7	
t-1	79	24	39	47.3	
t	77	20	40	45.7	0.01

Table 1: Example of Ambiguity in Google Searches. The body of the table presents the search intensity of each word.

vere, this index should serve as a proxy for the pandemic's severity. When included as a control variable in the Event Study methodology in Tables 4 and 5, the index turns out to be statistically insignificant. However, in the Panel Data specification in Table 6, columns (7) and (8), the stringency index exhibits a positive sign, which suggests that around government announcements, the prevalence of more strict lockdown policies induces more pandemic-related internet searches.

Concerning the keywords, we do not label the query as positive or negative because we believe that most of these words have a negative connotation.

Furthermore, beyond your original comment and related to the other sentiment proxy, based on Twitter, we also detect that the strictness of lockdown policies implemented by the government has in fact a negative impact on overall market sentiment, as reported in columns (3) and (4) of the same table.

C5: 2.3- The authors should provide a more comprehensive robustness analysis of the baseline results. It would be interesting to run a robustness check controlling for the weight of the different sectors in each region (municipalities if possible).

A5: Thank you for your comment. As a robustness check, we consider an alternative sorting of municipalities' socioeconomic status, based on *Municipal Income*. Results for stock market reactions are presented in Table 3. We obtain similar results to those obtained for the *MPI* sorting. Regarding the 12% threshold used in the baseline specification of sentiment analysis, this figure corresponds to the proportion of people belonging to the $ABC1$ socioeconomic segment for 2018, according to the Association of Market Researchers and Public Opinion of Chile. In section 4, Tables 3, 4, and 5, we perform a sensitivity analysis and present our results for different wealth cohorts, not only for the *ABC1* segment of the population.

Regarding your comment that it would be interesting to run a robustness check controlling for the weight of the different sectors in each municipality, there is certainly socioeconomic heterogeneity within municipalities. However, as mentioned in the revised version of the paper, they are the minor administrative division in Chile. The health authorities impose quarantines

in such a way that confinement affects the whole universe of inhabitants of each municipality. Accordingly, even if we had a more refined socioeconomic segmentation of people inside municipalities, that we don't, we would not be able to make use of it.

C6: 2.4- The authors should provide a more comprehensive of the economics factors behind the story they are telling..

A6: Thank you for your comment. As you will notice, we have made significant changes to the paper. Throughout the revised version, we have tried to provide reasonable explanations for the observed phenomena our work is concerned about.

C7: 2.4- - The organization of the paper should be improved. In particular, the authors should consider a section summarizing all the correlations presented in Section "Social Sentiment Reactions to Quarantine Announcements".

A7: Thank you for your comment. We have thoroughly revised the organization of the paper to provide a clear motivation for our research question. In the revised version, in Section 4, we present all the empirical results we entertain in our work.

C8: 2.4- - The authors should explain the differences between both exercises. It seems that they use the same data set but changing the order of causality. If this is the case the rationality of this methodology should be explain in order to better understand the results.

A8: Thank you very much for your comment. The "Sentiment Controls" included in Eq. (13) in the original version of the document were the sentiment proxies used later on in the sentiment analysis. In the revised version of our work, we do not include these controls in Eq. (5). In any case, when originally included as controls, these variables turned out to be statistically insignificant.

REVIEWER 6:

C1: 1) Include some official sources regarding information mentioned in the

second and third paragraphs of the introduction.

A1: Thank you for your comment. Your suggestion has been addressed in the revised version of the paper.

C2: 2) Include the link of INE and CASEN survey

A2: Thank you for your comment. Your suggestion has been addressed in the revised version of the paper.

C3: 3) Include an official source about the 25 government announcements

A3: Thank you for your comment. Your suggestion has been addressed in the revised version of the paper.

C4: 4) Chile was the only country to implement a dynamic quarantine? Otherwise, you could comment/compare to other countries

A4: Thank you for your comment. Other countries have implemented limited rather than country-wide lockdowns. For instance, Italy and Spain imposed country-wide lockdowns only after the failure of limited quarantines to control the pandemic. In any case, it should be noted that the Chilean strategy has been quite different. While in a limited quarantine typically certain services, schools and non-essential workplaces business were closed, the dynamic quarantine scheme imposes general restrictions at certain geographical zones (municipalities). As mentioned in the revised version of the paper, municipalities are the minor administrative division in Chile, and the health authorities impose quarantines in such a way that confinement affects the whole universe of inhabitants of each municipality.

C5: 5) Draw an object process diagram about the analyzes realized in the methodology section. It will help visualize all the steps

A5: Thank you for your comment. We have included Figure 4, a process diagram, to schematically show the analyzes carried out in the methodology section.

C6: Why you did not test classification algorithms for Twitter classification?

A6: Thank you for your comment. We do not label twitter sentiment as positive or negative. We made use of sentiment as a continuous variable (see Figure 3). Therefore, the methodological approach in this study does not require a classification algorithm.

C7: 7) Highlit with bold the most important values in table 1 / provide some visual graphic. Since it presents many values it is difficult to analyze them

A7: Thank you for your comment. You are right. It isn't easy to interpret the results in Table 2 without knowing which factors led to the corresponding stock market or sentiment reaction on any given date. That is why we explain the magnitude and signs of these reactions through the estimations of equations (5), (14), and (15). In any case, the point of Table 2 is to show the existence of heterogeneity in both stock market reactions and sentiment responses upon government announcements.

C8: 8) Include a discussion section comparing your results with related work. I miss some comparisons with other works.

A8: Thank you for your comment. In the Introduction and in the Conclusions we now emphasize how our work contributes to the existing literature and highlight its place in the literature concerned with the causal effect between lockdowns and the population overall well-being during the pandemic. Also, the literature review has been expanded. To keep the manuscript at a reasonable length, in Section 2, Table 1, we present a brief review of recent works related to sentiment analysis and the place of our work in that context.

C9: 9) Add challenges and limitations in the conclusion section

A9: Thank you for your comment. Limitations regarding our sentiment proxies, methods and empirical strategies are presented in the Conclusions.

C10: 10) some references have doi and others don´t

A10: Thank you for your comment. Your suggestion has been addressed in the revised version of the paper.

We thank the anonymous Reviewers for their comments and efforts to improve the paper.

Sincerely,

Fernando Díaz
Pablo A. Henríquez
