# Supplementary material for: Social sentiment segregation: Evidence from Twitter and Google Trends in Chile during the COVID-19 dynamic quarantine strategy
Source: PLoS One. 2021 Jul 13;16(7):e0254638. doi: 10.1371/journal.pone.0254638 (PMC8277056; doi:10.1371/journal.pone.0254638)
Supplement: Attachment — Submitted filename: Response to Reviewers.pdf [file pone.0254638.s006.pdf]

Santiago, Chile, June 10th, 2021

Dear,
Stefan Cristian Gherghina, Academic Editor,
PLOS ONE

We would like to thank you for your letter on 2 June 2021 with the editorial decision of the manuscript 'Social Sentiment Segregation: Evidence from Twitter and Google Trends in Chile during the COVID-19 Dynamic Quarantine Strategy' (Díaz,F.,Henríquez, P.A.).

We have completed the revision of the manuscript according to the comments of the Academic Editor (AE) and the Reviewers.

In this letter, the comments (C) by the AE and Reviewers are reproduced and a detailed answer (A) is given concerning the changes made in the manuscript.

AE:
C1: The revised version of the manuscript improved in a positive manner, but further revisions regarding empirical outcomes' discussion and concluding remarks are required.

A1: Thank you, we have addressed all the minor changes requested.

REVIEWER 1:
C1: In my previous report, I had raised concerns regarding measurement, interpretation, and exposition. The authors revised the paper along several dimensions, taking into account many of my concerns. Overall, the paper is now better written and more convincing. However, I believe some points are left unaddressed.

1) I still feel unsure about the choice of 12% as a threshold to define wealthy municipalities. The authors conduct some robustness around this number, which is certainly reassuring. However, the way this threshold is chosen is still puzzling to me. My understanding is that 12% is the share of

people in ABC1. Is this share “geographical” in any way (besides the obvious fact that richer people will tend to reside in richer municipalities)? In other words, how are the 12% of people belonging to ABC1 connected to the 12% of people living in the richer municipalities?

A1: Thank you for your comment. Your comment seems to be twofold. In the first place, the 12% figure corresponds to the proportion of people belonging to the ABC1 socioeconomic segment for 2018, according to the Association of Market Researchers and Public Opinion of Chile. In any case, and following your comments to the previous version of the paper, in Tables 4, 5 and 6 we present our results for different aggregations of the population, based on the municipalities sorting. In the second place, regarding your concern about the connection between the SES of the whole municipality and the variability in the SES of their inhabitants, we admit that there is undoubtedly intramunicipal socioeconomic heterogeneity. In this sense, you are right. When we consider, for instance, the top 5 wealthier municipalities in Table 4 (or 5 or 6), we assume that the whole population that they aggregate (4.68% of the country population) belongs to the richest segment, disregarding the fact that there is undoubtedly inhabitants of those municipalities that do not belong to it. To clarify this point, we add a new table (Table 2) and provide the following explanation (lines 187 to 196):

“Since lockdowns affect all the inhabitants of a given municipality, it should be noted that to obtain the approximate number of people from a given SES that is confined upon government announcements, we assume that the whole population of that municipality belongs to the same segment, disregarding the SES heterogeneity that their inhabitants naturally have. However, a quick look at Table 2 reveals that wealthy municipalities exhibit a much lower variability in the MPI of their inhabitants than non-wealthy ones, where variability is defined as the range of the poverty index for a given municipality. In this sense, since wealthy municipalities are far less heterogeneous, identifying the wealthiest population through the municipality they reside in does not seem particularly troublesome.”

This information can also be seen graphically. As shown in the figure below, wealthier municipalities exhibit a much lower variability in the SES composition of their inhabitants than non-wealthy ones.

C2: 2) The authors interpret their findings in terms of degree of “segregation” in financial markets and internet platforms. I still do not see why

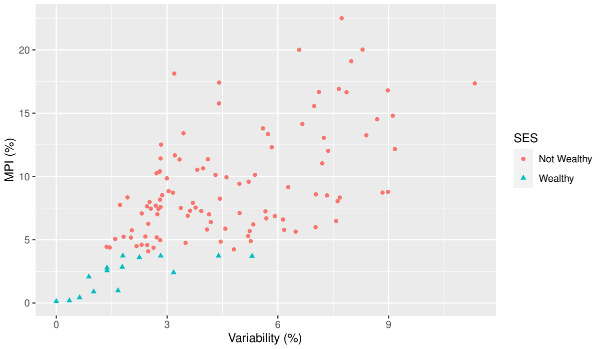

Figure 1: SES variability

the response to lockdown decisions is necessarily informative of the degree of segregation by socioeconomic status. There can be other reasonable explanations for why the financial market reacts more strongly to lockdowns in richer municipalities. For example, it is possible that lockdowns in richer municipalities have a more pronounced impact on the overall economy, or that publicly traded firms tend to be located in richer municipalities, and are more likely to be affected by lockdowns in those municipalities. One could build analogous arguments to explain the heterogeneity in the results for the other measures. Alternative explanations should at least be considered, mentioned, and discussed. If the authors believe that the heterogeneity in the estimated effects for CASA and CASVA can only be reconciled by postulating different degrees of socioeconomic segregation for Twitter and Google users, this should be made explicit. It is possible that I am simply missing what the authors mean by "socioeconomic segregation" in this setting. In this case, I encourage the authors to provide more guidance to the reader and make the definition of this concept as clear as possible.

A2: Thank you very much for your insightful comment. Regarding stock market reactions to government announcements, following your suggestion, in the previous version of our work we de-emphasized the observed stock market phenomenon as a central finding in our work and explained that the stock market analysis was performed mainly to validate the proposed *SES* sorting among municipalities.

In line 471 of the previous version, we argue that as richer cohorts are considered for the changes in the number of people under lockdown, the pre-

dicted abnormal returns to the stock market are higher in magnitude, and that changes in the total population cannot explain stock market reactions to such announcements. We interpret this result as a consequence of socioeconomic segregation. In fact, the income inequality in Chile is the highest among the OECD countries. According to the World Inequality Database (`https://wid.world/`), as of year-end 2018, the top 10% wealthiest population accounts for 60.4% of the total income of the country. Following this line of reasoning, in line 476 of the previous version, we stated that:

"This result is important for at least two reasons. First, it documents the high level of economic segregation that the country exhibits based on an observable market phenomenon. Second, it validates our proposed wealth ranking, which will also be used in the analysis of sentiment responses to government announcements below."

But, despite our arguments, you are right in the sense that there might be other explanations for why the financial market reacts more strongly to lockdowns in richer municipalities. Consequently, and acknowledging your observation, we have added the following in line 478:

"Recognizing that there might be several reasons why such a phenomenon could be observed, it strongly suggests a high level of wealth concentration among the richer population. In fact, according to the World Inequality Database (`https://wid.world/`), as of year-end 2018, the top 10% wealthiest population accounts for 60.4% of the total income of the country."

In any case, since it is not a central issue in our work, and we use such result to show that our proposed wealth sorting makes sense, we have removed our mentions to stock market and socioeconomic segregation. We have changed the text, and it now reads as follows in line 485:

"This result is important because it validates our proposed wealth ranking, which will also be used in the analysis of sentiment responses to government announcements below."

C3: 3) I agree with the choice of including the "Stringency Index" as a control. However, I still believe that controlling for CAR in Tables 4 and 5 makes the inference worse. The stock market response is an outcome of the lockdown announcements, so its inclusion as a control makes it harder to evaluate the actual treatment effect of the announcement on CASA and CASVA. In Angrist and Pischke (2009)'s terminology, this is a "bad control", in the sense that it is itself an outcome of the experiment.

A3: Thank you very much for your insightful comment. You are completely right. In any case, results remain nearly unchanged when the stock market reactions are not included as controls in the econometric specification, probably because of its lack of significance. In the following table, we report the results for the *Cumulative Abnormal Sentiment Activity* for the (-1,+1) window. Comparing these results to those reported in Table 5, the estimated coefficients for the change in population change very little, both in magnitude and statistical significance. Furthermore, just as in the original table, we obtain a nearly monotonically decreasing magnitude of market sentiment responses to government announcements as we move from the wealthiest municipalities in column (1) to the whole population in column (6).

	Twitter $(-1,+1)$					
	Cumulative Abnormal Sentiment Activity - *MPI* Sorting					
	(1)	(2)	(3)	(4)	(5)	(6)
Δ Top 5 (4.68%)	-4.1057^{+}					
	(2.7041)					
Δ Top 10 (9.57%)		-3.0879^{*}				
		(1.7624)				
Δ Top 15 (12.76%)			-3.1121^{**}			
			(1.5814)			
Δ Top 20 (18.20%)				-2.6485^{*}		
				(1.4738)		
Δ Top 24 (22.37%)					-2.5709^{*}	
					(1.5171)	
Δ Total Pop.						-1.7508^{*}
						(0.9908)
SI	−0.0447	−0.0386	−0.0408	−0.0555	−0.0568	−0.0475
	(0.0629)	(0.0523)	(0.0496)	(0.0585)	(0.0560)	(0.0410)
Observations	25	25	25	25	25	25
R^2	0.4969	0.6674	0.7179	0.6748	0.6457	0.5240

Note: Bootstrapped Std. Errors + p<0.15; * p<0.1; ** p<0.05; *** p<0.01

Table 1: Sentiment (Twitter) vs. Changes in Confined Population (Population in Millions)

C4: 4) While the quality of the writing is now significantly improved compared to the previous version, there are still inconsistencies in the notation and typos. Here are a few typos I have found:

- In Equation (3), the sub-index “i” is in the right-hand-side, but not in the left-hand-side.
- In Equation (5), the sub-index “i” refers to “cohort”, while in Equation (3) it refers to the stock market index.
- The notation in Equations (10) and (12) displays a similar inconsistency.
- I believe “columns (7) to (8)” at line 502 should be “columns (7) to (12)”.
- At line 644, “expected sings” should be “expected signs”.

References

Angrist, J. and Pischke, J.-S. (2009). “Mostly harmless econometrics: an empiricists guide”. Princeton: Princeton University Press.

A4: Thank you for taking the time to read our article in detail. All your suggestions have been properly addressed.

REVIEWER 2:
C1: I would like to congratulate the authors on the substantial revision of their manuscript. You have adequately addressed my earlier comments and suggestions.

I do have two minor comments left:

A1: Thank you for your comments, as you will see below we have addressed all of them.

C2: - While I laude the authors in implementing an additional estimation (the DiD 'like' approach) to accommodate a larger sample size, 325 is still rather minimal. Hence, I advise the authors to stress this caveat explicitly in the final version of the paper.

A2: Thank you for your comment. In line 585, we have included the following:

“It should be noted, however, that the sample size achieved is still rather

small, and results should be interpreted in light of this limitation."

C3: - Regarding my earlier comment 3.a, I understand that the municipal level is the most granular available. Moreover, I also understand that restrictions were applied to the municipality as a whole. Nevertheless, this does not fully address my concern that inferring general socioeconomic correlations based on the rank of an entire municipality is somewhat of a stretch, because after all - as acknowledged by the authors - the municipal level is compounding large heterogeneities. Therefore, I urge the authors to come up with a more satisfactory explanation for why their conclusions are immune to this. Otherwise, the conclusions should be phrased in less strong terms. Simply not having the data seems rather unsatisfactory.

A3: Thank you for you comment. You are right. There is undoubtedly intramunicipal socioeconomic heterogeneity, but we believe that our approach overtakes this caveat, given the low heterogeneity that wealthy municipalities exhibit in the variability of their MPI. Please, refer to Figure 1 above, Table 2 in the article and answers **A1** and **A2** to Reviewer 1, that expressed basically the same concerns as you about this issue.

REVIEWER 3:
C1: Dear authors The revised manuscript considered all correction comments within the current version regards

A1: Thank you for all your comments to improve the paper.

REVIEWER 4:
C1: The authors addressed all my comments and concerns, I believe the manuscript now is suitable for publication

A1: Thank you for all your comments to improve the paper.

REVIEWER 5:
C1: The authors made substantial changes to the manuscript, in accordance with the Reviewers' comments, the text is more readable. I have only a minor comment regarding ambiguity. The search volume intensity includes the following 19 terms: corona, OMS (WHO), virus, COVID-19, SARS, MERS, epidemia (epidemic), pandemia (pandemic), síntoma (symptom), infectado

(infected), propagación (spread), brote (outbreak), distanciamento social (social distancing), restricción (restriction), cuarentena (quarantine), suspender (suspend), viajar (travel), encierro (lockdown) and mascarilla (face mask). The authors claimed that even if there is ambiguity in the search of pandemic related words, this goes against the effect they intent to quantify. The reason is that if the search intensity of key words that are subject to ambiguity do not change around government announcements (because they are in fact ambiguous), the statistic in Eq 9 in the revised version of the paper would be downward biased. My concern in that some of these terms may change without any relation with government announcements. Moreover, the change may be on the wrong direction. This is the case for "suspender" that can be use against different backgrounds far away from the COVID-19 crisis.

A1: Thank you very much for your comment. You are right, there might be ambiguity in the terms used to construct our proposed Google Sentiment measure. In line 716-718, we have included the following:

"Lastly, some of these 19 terms may change, in either direction, without a direct relation with government announcements."

We thank the anonymous Reviewers for their comments and efforts to improve the paper.

Sincerely,

Fernando Díaz H.
Pablo A. Henríquez
