# Supplementary material for: Social sentiment segregation: Evidence from Twitter and Google Trends in Chile during the COVID-19 dynamic quarantine strategy
Source: PLoS One. 2021 Jul 13;16(7):e0254638. doi: 10.1371/journal.pone.0254638 (PMC8277056; doi:10.1371/journal.pone.0254638)
Supplement: Attachment — Submitted filename: PONE-D-21-01436.docx [file pone.0254638.s004.docx]

Referee Report: “Twitter and Google Trends Sentiment in a highly segregated economy: The COVID-19 Case in Chile”

Manuscript Number: PONE-D-21-01436

1 Overwiew

This paper analyses social media and stock market reactions to the government announcements made by the Chilean health authority in the context of the COVID-19 pandemic. Taking advantage of the heterogeneity in the size and socioeconomic characteristics of the country's municipalities, the authors document a socioeconomic segregation phenomenon, both in the stock market and among Twitter users. The main findings can be summarized as follows: First, they provide evidence that observed stock market abnormal returns can be explained by the number of people from the wealthiest segment of the population that enters or exits confinement. Second, social sentiment based on Twitter tends to be more pessimistic, the higher the number of the wealthiest population that is quarantined.

2 Comments

Overall I enjoyed reading the paper and I consider the topic to be relevant, although certain aspects need to be improved. I have several suggestions which are aimed at clarifying the story the author is telling. These are not in any particular order.

2.1- The proposed Google Sentiment measure is constructed using 19 terms. In general, queries based on keywords are language-specific and subject to ambiguity. The authors should clarify why using keywords instead of topics

2.2- Related to the previous point, the authors argue that “the higher the value of the ASV in a given day t, the higher the population's attention to the outbreak of the Coronavirus on that day”. However, this argument is not taking into the relationship between intensity and severity. Should each query be labelled as positive or negative? The authors should clarify this point

2.3- The authors should provide a more comprehensive robustness analysis of the baseline results. It would be interesting to run a robustness check controlling for the weight of the different sectors in each region (municipalities if possible).

2.4- The authors should provide a more comprehensive of the economics factors behind the story they are telling..

2.4- - The organization of the paper should be improved. In particular, the authors should consider a section summarizing all the correlations presented in Section “Social Sentiment Reactions to Quarantine Announcements”.

2.4- - The authors should explain the differences between both exercises. It seems that they use the same data set but changing the order of causality. If this is the case the rationality of this methodology should be explain in order to better understand the results.
